# Dynamic Expression of the Translational Machinery during *Bacillus subtilis* Life Cycle at a Single Cell Level

**DOI:** 10.1371/journal.pone.0041921

**Published:** 2012-07-25

**Authors:** Alex Rosenberg, Lior Sinai, Yoav Smith, Sigal Ben-Yehuda

**Affiliations:** 1 Department of Microbiology and Molecular Genetics, Institute for Medical Research Israel-Canada (IMRIC), The Hebrew University, Hadassah-Medical School, The Hebrew University of Jerusalem, Jerusalem, Israel; 2 Genomic Data Analysis Unit, The Hebrew University- Hadassah Medical School, The Hebrew University of Jerusalem, Jerusalem, Israel; Baylor College of Medicine, United States of America

## Abstract

The ability of bacteria to responsively regulate the expression of translation components is crucial for rapid adaptation to fluctuating environments. Utilizing *Bacillus subtilis (B. subtilis)* as a model organism, we followed the dynamics of the translational machinery at a single cell resolution during growth and differentiation. By comprehensive monitoring the activity of the major *rrn* promoters and ribosomal protein production, we revealed diverse dynamics between cells grown in rich and poor medium, with the most prominent dissimilarities exhibited during deep stationary phase. Further, the variability pattern of translational activity varied among the cells, being affected by nutrient availability. We have monitored for the first time translational dynamics during the developmental process of sporulation within the two distinct cellular compartments of forespore and mother-cell. Our study uncovers a transient forespore specific increase in expression of translational components. Finally, the contribution of each *rrn* promoter throughout the bacterium life cycle was found to be relatively constant, implying that differential expression is not the main purpose for the existence of multiple *rrn* genes. Instead, we propose that coordination of the *rrn* operons serves as a strategy to rapidly fine tune translational activities in a synchronized fashion to achieve an optimal translation level for a given condition.

## Introduction

*rrn* operons in bacteria consist of three genes encoding the rRNA molecules: 16 S, 23 S and 5S rRNAs. 16S rRNA comprises the small ribosomal subunit, while the latter two rRNA molecules are part of the large ribosomal subunit [1]. The *rrn* operon copy number varies among bacterial genomes from a single copy in bacteria such as *Mycobacterium tuberculosis* to as many as 15 copies in others, such as *Clostridium paradoxum* [1]–[3]. It has been proposed that the multiple copies of *rrn* operons are required to achieve an efficient growth, since rRNA was shown to constitute approximately 80% of total RNA in rapidly growing *Escherichia coli* (*E. coli*) cells [4]. However, short doubling times are not exclusively restricted to organisms harboring multiple *rrn* operons. For example, *Pyrococcus furiosus* possesses only one *rrn* operon but has a relatively short doubling time of 37 minutes [5]. Furthermore, deleting two out of the seven *E. coli rrn* operons had only a slight effect on maximal growth rates, though the lag time was significantly extended upon transfer into fresh medium [6]–[8]. In light of these observations, it was also suggested that multiple *rrn* operons are advantageous in changing environmental conditions, when rapid adjustment to nutrients and temperature is beneficial [6]–[10].

The molecular mechanism of *rrn* transcription regulation has been extensively studied over the last 60 years, with the Gram negative bacterium *E. coli* serving predominantly as the model organism [1], [11]. In general, *rrn* expression is regulated at the level of transcription initiation and corresponds to nutrient availability. Hence, the rRNA level correlates with exponential growth, decreasing rapidly upon transition from higher to lower growth rates [12]–[16]. Interestingly, under starvation the linkage between *rrn* transcription and nutrient availability is perturbed leading to the production of excess amounts of rRNA [17], [18].

The Gram positive bacterium *B. subtilis* contains 10 *rrn* operons that appear to differ in their regulation from those of *E. coli* [19]. The regulatory dissimilarities between the two evolutionary distinct species can be attributed, at least in part, to different elements within the *rrn* promoter sequences [19]–[21]. Further, *B. subtilis* possesses a unique life cycle comprising various phases of growth and development, including vegetative growth, sporulation and germination. The transition from one mode of development to another is driven by nutrient availability, which is sensed by the bacterium. Upon starvation *B. subtilis* initiates sporulation, a process that leads to production of a resilient cell type called a spore. At the beginning of sporulation, a reduction in the overall rRNA synthesis takes place [22]–[25]. However, reducing *rrn* copy number perturbs sporulation, suggesting that a threshold of rRNA level is necessary for the proper execution of the process [26]. In the following stages of sporulation, a polar septum is formed, dividing the developing cell into two unequal progenies, the small forespore compartment that becomes the mature spore and the larger mother-cell, which nurtures the developing spore. Within approximately 8 hours, the mother-cell lyses, and the generated spore is discharged [27], [28]. Spores constantly monitor their environment via germination receptors located in the spore inner membrane. When conditions are favorable, the spore undergoes germination and outgrowth restoring a vegetative life form [29], [30]. The rate of rRNA production is low during germination, but increases significantly upon initiation of outgrowth [31].

To characterize the dynamics of the translational machinery during the life cycle of *B. subtilis* and investigate differential regulation, if any, of the various *rrn* operons, we systematically monitored the expression patterns of 7 *rrn* promoters and additional translational components throughout growth, sporulation and germination at the single cell level. We describe the activity, dynamics and the expression variability of each element during the different phases of *B. subtilis* growth and development.

## Results

### Monitoring Expression of Translational Components during Growth in Rich Medium

The genome of *B. subtilis* contains ten ribosomal *rrn* operons [(*rrnO*, *A*, *J*, *W*, *I*, *H*, *G*, *E*, *D*, *B*) with the *rrnJ*–*rrnW* and *rrnI*–*rrnH*–*rrnG* grouped as clusters] (Fig. S1A), the expression of which is considered the rate limiting step for ribosome production [32]. To investigate the activity of the different *rrn* operons at a single cell level, we constructed an array of *B. subtilis* strains, each harboring a *gfp* fusion to a different *rrn* promoter. Based on work of Koga and colleagues that characterized the *rrn* promoters [33], seven such strains were generated representing, the major *rrn* operons (Fig. S1B). Testing the suitability of GFPmut2 as a reporter for measuring translational dynamics revealed the protein half-life to be approximately 5.6 hrs (Figure S2), which is lower than previous studies that utilized GFP as a reporter for genome wide expression analyses in *B. subtilis* [34]–[36]. Importantly, insertion of the fusions had no significant effect on cell growth and division (Figure S1C). GFP signal emanating from these strains was then measured by fluorescence microscopy to assess the activity and the dynamics of the translational components at different growth phases.

**Figure 1:**
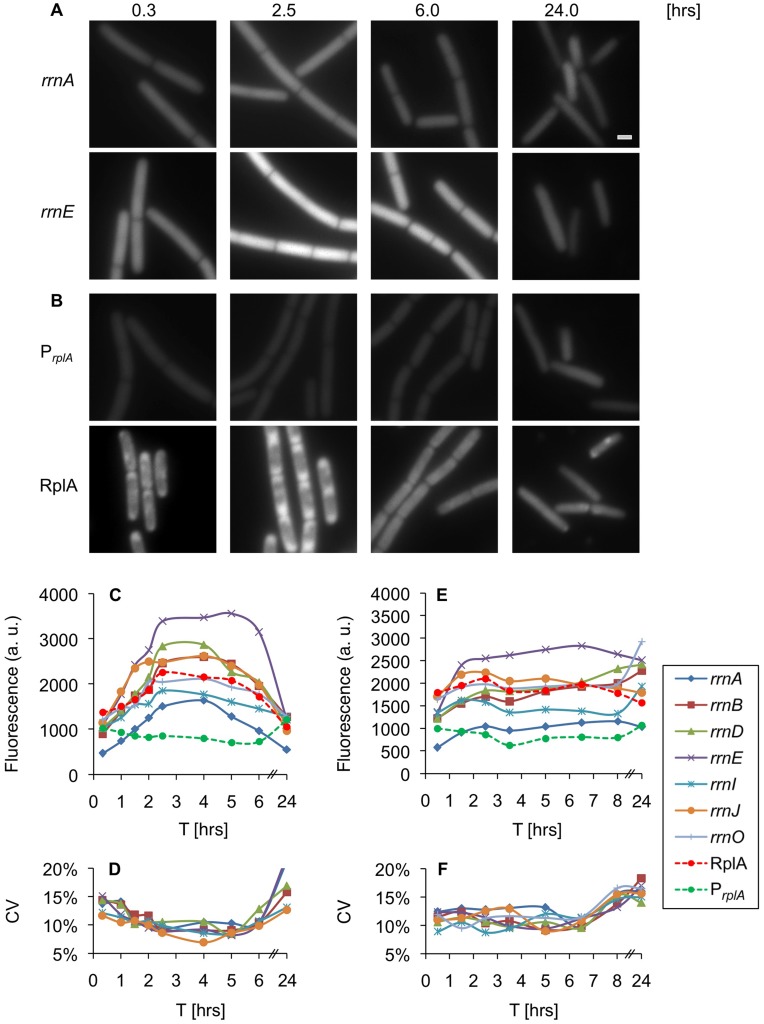
Expression of ribosomal components is dynamic during vegetative growth. (**A–B**) Strains carrying *P_rrnA_-gfp* (AR13) (A-*rrnA*), *P_rrnE_-gfp* (AR16) (A-*rrnE*), *P_rplA_-gfp* (AR25) (B-*P_rplA_*), *rplA-gfp* (AR5) (B-RplA) were grown in rich medium (CH). Samples were taken at the indicated time points [hrs] and the GFP signal monitored using fluorescence microscopy. All fluorescence images were normalized to the same intensity range. Of note, the localization of RplA-GFP was consistent with previous results [78]. Scale bar corresponds to 1 µm. (**C**) Strains carrying *P_rrn_-gfp* (*rrnO, A, B, D, E, I, J*), *P_rplA_-gfp* or *rplA-gfp* were grown in rich medium (CH). Samples were taken at the indicated time points [hrs] and the GFP signal monitored using fluorescence microscopy. The data represent the average of three independent biological repeats. Fluorescence from at least 60 cells was measured and averaged for each time point, and is shown in arbitrary units (a.u.) (see Materials and Methods). (**D**) Variability of GFP intensity among single cells carrying the indicated *P_rrn_-gfp* reporter at the different time points described in (C). Coefficient of Variability (CV) was calculated as SD divided by mean (see Materials and Methods). (**E**) Strains carrying *P_rrn_-gfp* (*rrnO, A, B, D, E, I, J*), *P_rplA_-gfp* or *rplA-gfp* were grown in minimal (S7) medium and samples processed as in (C). (**F**) Variability of GFP intensity among single cells carrying the indicated *P_rrn_-gfp* reporter at the different time points described in (E). Coefficient of Variability (CV) was calculated as SD divided by mean (see Materials and Methods).

GFP expression was monitored in individual cells harboring the various *rrn* reporters growing in rich medium. The examined reporters exhibited different activity levels however; their dynamic profiles were similar (Figure 1A and 1C). The transcriptional activity of all seven *rrn* promoters was upregulated during the initial growth stages, reaching maximal activity in mid logarithmic phase (Figure 1A and 1C, 2.5 hrs). These high expression levels were maintained almost constant until entry into stationary phase (Figure 1C, 4 hrs), during which the promoters activity was down regulated. Finally, the promoters reached their minimal expression level in deep stationary phase (Figure 1A and 1C, 24 hrs). Evaluating the transcriptional variability of the *rrn* promoters at a single cell resolution revealed an inverse correlation between variability and expression level, with maximal variation obtained during deep stationary phase (Figure 1D). Thus, lower levels of *rrn* promoter activity were associated with greater transcriptional heterogeneity among single cells.

To correlate the transcriptional activity of the *rrn* promoters with that of ribosomal protein constituents, strains carrying either transcriptional or translational *gfp* fusions to a gene encoding the key ribosomal component RplA (ribosomal protein L1) were constructed (Figure S1A). In contrast to the *rrn* promoters, the *rplA* promoter activity was constant throughout the different growth phases showing an increase during deep stationary phase (Figure 1B and 1C). However, the protein level correlated positively with the expression pattern observed for the *rrn* promoters (Figure 1B and 1C). These data corroborate previous results, indicating that ribosomal protein expression is regulated primarily post-transcriptionally [37], [38].

Taken together, in rich medium the expression of all tested *rrn* promoters is growth phase dependent. The heterogeneity among single cells is low in logarithmic phase when rRNA expression is maximal and most evident during stationary phase when the expression is minimal. As expected [38], the levels of the ribosomal protein RplA parallel rRNA expression, implying synchronous regulation of the different ribosomal components.

### Monitoring Expression of Translational Components during Growth in Poor Medium

Since the translational activity is regulated by growth rate, we now wished to explore the effect of nutrient limitation on *rrn* promoter activity. When cells were grown in minimal medium, the transcriptional activity of the *rrn* promoters was reduced by an average of 30%, compared with the level monitored in rich medium (Figure 1C and 1E). The activity profile of the *rrn* promoters during growth showed an initial boost during logarithmic phase, after which it remained rather constant until reaching stationary phase (Figure 1E, 0–4 hrs). Surprisingly, unlike the pattern observed in rich medium, the transcriptional activity of the *rrn* promoters did not drop dramatically during stationary phase. Four of the investigated promoters, *rrnB,D,I*,*O* displayed a significant increase in their expression during deep stationary phase, while the remaining three promoters *rrnA*,*E*,*J* exhibited a mild decrease (Figure 1E, 24 hrs). To further corroborate that the observed increase in *rrnB,D,I*,*O* fluorescence emanates from an actual promoter activity, we performed fluorescence recovery after photobleaching (FRAP) experiments. When cells residing in deep stationary phase were irradiated, the fluorescence signal was largely restored 20 min post bleaching (Fig. S3), indicating that the cells retain substantial metabolic activity at this phase. The *rrn* upregulation may represent an attempt to counteract the degradation of rRNA not incorporated into ribosomes, a phenomenon previously reported to occur in *E. coli* cells grown under limited nutrient resources [39], [40]. Indeed, analysis of the rRNA derived from deep stationary phase cells revealed a reduction in the amount of intact rRNA and the appearance of fragmented rRNA species (Fig. S4A). In line with this view, similarly to the *rrn* promoters, the activity of the *rplA* promoter was increased during this phase; however, RplA-GFP expression was down regulated (Figure 1E). The lack of correlation between RplA-GFP production and transcriptional activity of the *rplA* promoter may derive from a shorter protein/mRNA half-life during stationary phase.

Following the activity profile of the different *rrn* promoters upon transfer of stationary cells to fresh medium revealed an interesting phenomenon. The promoters *rrnB,D,I*,*O*, displaying high activity during deep stationary phase showed a decreasing pattern upon suspension in fresh medium. However, *rrnA*,*E*,*J* and RplA originally exhibiting a reduction, increased activity during this transition (Fig. S4B). Accordingly, a global decline in rRNA degradation products was monitored, and the total rRNA amount resumed its early logarithmic phase level (Fig. S4A). These observations suggest that the translational machinery readjust its levels, responding to the new conditions.

**Figure 2:**
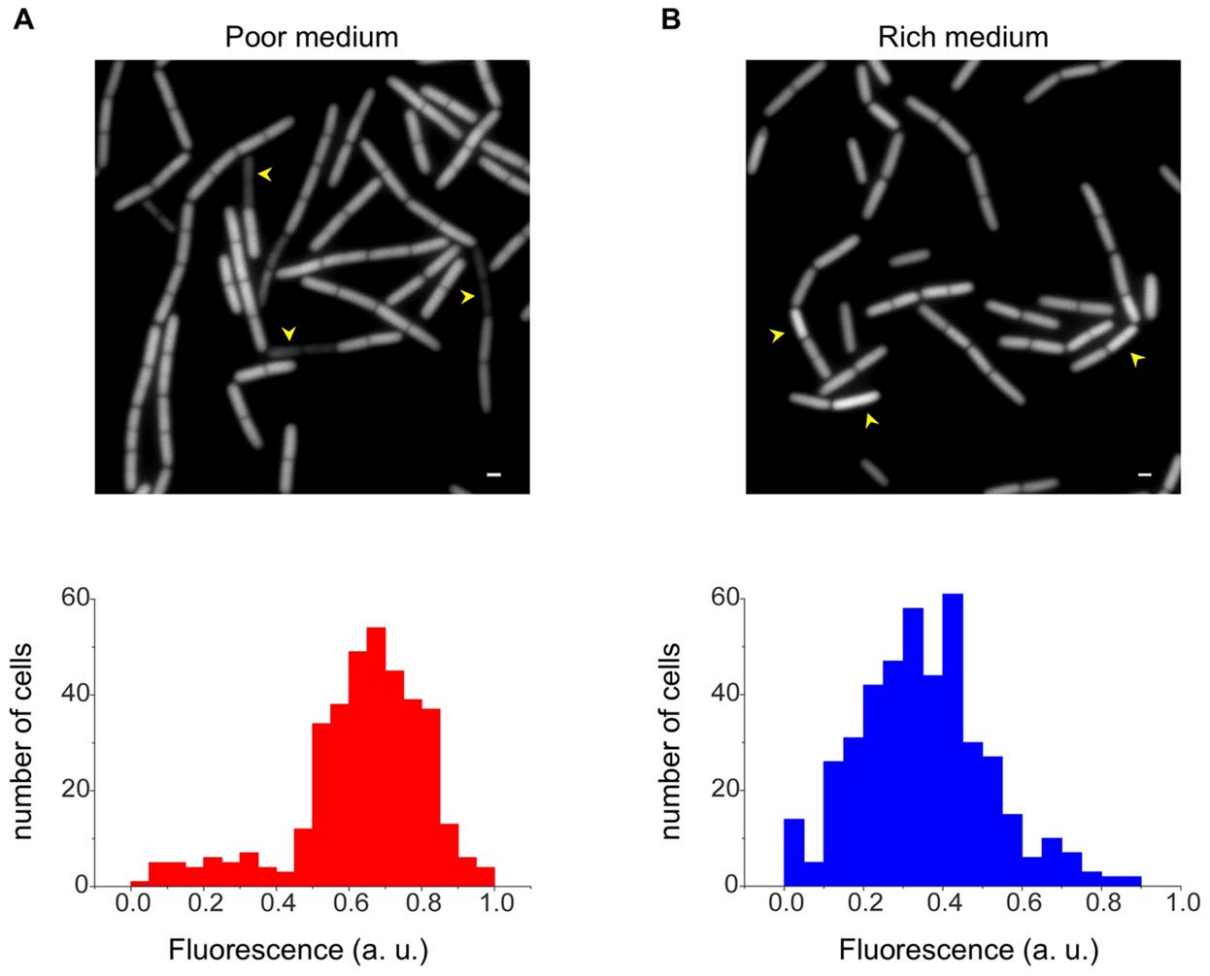
Bimodal *rrn* activity during stationary phase. Cells carrying the P*_rrnO_-gfp* (AR17) were grown in poor medium (S7) (**A**) or rich medium (CH) (**B**). Shown are GFP fluorescence images acquired from stationary phase cells (upper panels). Normalized fluorescence distribution of at least 100 individual cells (lower panels) is scored for each phase and is shown in arbitrary units (a.u.). Arrowheads highlight cells displaying either low (A) or high (B) *rrn* promoter activity, compared to the average level. Scale bars correspond to 1 µm.

Calculating the variability of *rrn* promoter expression among single cells revealed that, unlike the growth rate related pattern observed in rich medium, an almost constant level was apparent during vegetative growth (Figure 1F). This pattern is consistent with the relatively steady *rrn* promoter expression observed throughout this phase (Figure 1E). Notably, during stationary phase, when variability was largely increased, a subpopulation of cells exhibiting low fluorescence levels appeared in all tested promoters, giving rise to a distinctive bimodal expression pattern (Figure 2A). In comparison, cells reaching stationary phase in rich medium exhibited less variability, with a small subpopulation of cells displaying increase, rather than decrease, in *rrn* expression (Figure 2B). Following the stationary cells with low fluorescence levels in poor medium using time lapse microscopy revealed that they do not resume growth, at least for a period of a few hours, and even lyse upon transfer to fresh medium (Figure S4C), suggesting that the level of translational activity impact future cellular fates.

We conclude that translational dynamics is affected by nutrient availability, a phenomenon manifested during both exponential and stationary phases. Unlike the dynamic *rrn* expression exhibited by exponentially growing cells in rich medium, cells displayed little variation with almost constant *rrn* promoter activity when grown in poor medium. Moreover, in stationary phase *rrn* expression was maintained at high levels in poor medium, whereas in rich medium this activity was dramatically decreased.

### Differential Patterns of Translational Activity between Mother-cell and Forespore

**Figure 3:**
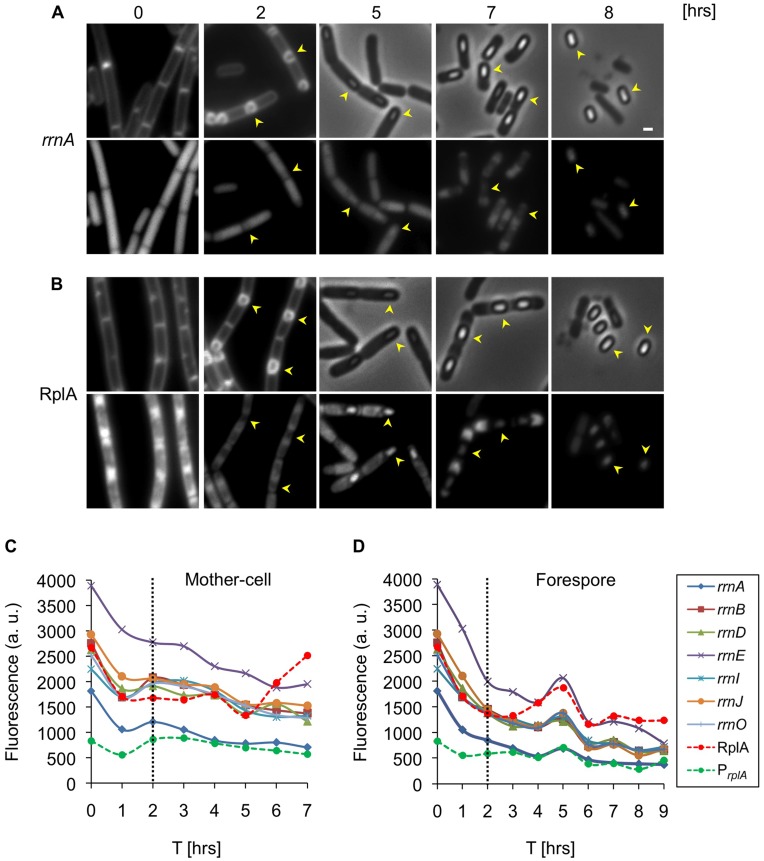
Translational machinery is differentially expressed within mother-cell and forespore. (**A–B**) Strains carrying P*_rrnA_-gfp* (AR13) (A) or *rplA-gfp* (AR5) (B) were induced to sporulate. Samples were taken at the indicated time points [hrs] and the GFP signal monitored using fluorescence microscopy. FM4–64 membrane stained cells (0 and 2 hrs) and phase contrast images (5–8 hrs) are shown in the upper panels and corresponding GFP fluorescence images (0–8 hrs) in the lower panels. Arrowheads designate the position of forespores. Scale bar corresponds to 1 µm. (**C–D**) Strains carrying *P_rrn_-gfp* (*rrnO, A, B, D, E, I, J*), *P_rplA_-gfp* or *rplA-gfp* were induced to sporulate. Samples were taken at the indicated time points [hrs] and the GFP signal from predivisional cells (0–2 hrs) and from mother-cell (C) and forespore (D) monitored using fluorescence microscopy. The data represent the average of 2 independent biological repeats. Fluorescence from at least 100 cells was measured and averaged for each time point and is shown in arbitrary units (a.u.) (see Materials and Methods).

To date, the transcriptional activity of *rrn* promoters during sporulation was measured at the population level using LacZ reporters [19], [33], [41], [42]. However, this approach does not allow the differentiation between sporulating and non-sporulating cells or between subcellular compartments. Hence, we utilized our single cell microscopy assay and the series of reporter strains to explore translational dynamics within the mother-cell and forespore compartments throughout sporulation. Strains harboring the *rrn* reporters were induced to sporulate, and samples were analyzed using fluorescence microscopy. All the analyzed strains displayed sporulation efficiency similar to that of the wild type strain. The progression through the early stages of sporulation was followed using a membrane stain that enables the forespore to be differentiated from the mother-cell. At later stages of sporulation, the presence of the forespore was determined by phase contrast microscopy. Concomitantly, GFP fluorescence emanating from the various reporters was visualized in and quantified from each of the cellular compartments. As early as one hour after suspension in sporulation medium, the transcriptional activity of the *rrn* promoters was significantly reduced (Figure 3A, 3C and 3D). Two hours post suspension, at the time the mother-cell and forespore emerged, an increase in *rrn* activity within the mother-cell was detected (Figure 3A and 3C). At the same time, the activity of the *rrn* promoters was reduced in the forespore compartment (Figure 3A and 3D). Subsequently, all tested *rrn* promoters exhibited continuously decreasing expression in the mother-cell (Figure 3C). In contrast, a sharp elevation in *rrn* activity was detected within the forespore five hours post induction of sporulation (Figure 3A and 3D). At this point, the activity of each *rrn* promoter in the forespore was equal to that observed in the mother-cell (Figure 3A). Finally, at later time points all tested *rrn* promoters in both cell types exhibited continuously decreasing expression until maturation of the spore and lysis of the mother-cell (Figure 3C and 3D). Thus, the mother-cell and forespore compartments undergo deferential *rrn* expression during the course of sporulation.

### Upregulation of the Translational Machinery within the Forespore

**Figure 4:**
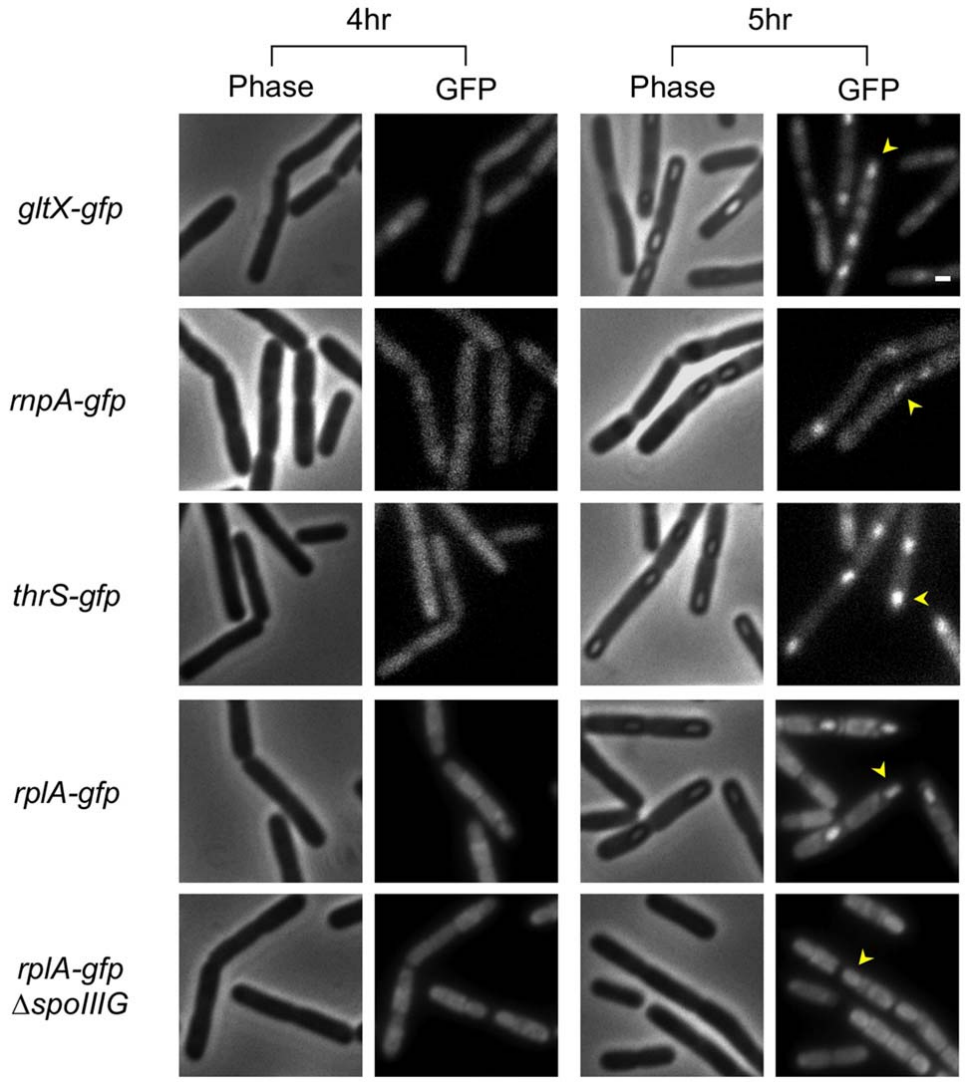
Expression of translational components increases transiently within the forespore. Strains carrying *gltX-gfp* (AR11), *rnpA-gfp* (AR10), *thrS-gfp* (AR9), *rplA-gfp* (AR5), or *spoIIIG::cat, rplA-gfp* (AR20) were induced to sporulate and samples taken at the indicated time points [hrs]. Shown are phase contrast images (left panels) and corresponding GFP fluorescence images (right panels). Arrowheads designate the position of forespores. Scale bar corresponds to 1 µm.

Having detected a transient increase in *rrn* promoter activity within the forespore compartment, we wished to explore whether this phenomenon is restricted to *rrn* promoters or can be measured for additional translational components. Initially, we monitored production of the RplA protein during sporulation. The pattern of RplA protein synthesis was similar to that of the *rrn* promoters, displaying a sharp increase within the forespore five hours into the process (Figure 3B, 3C, 3D). This increasing pattern is not likely to be a consequence of the appearance of the phase bright spore, as the fluorescence decreases at later stages while the brightness of the spore persists. Next, we constructed strains harboring GFP fusions to several translational components, namely GltX and ThrS, which are tRNA synthetases, and RNaseP, which is required for tRNA maturation [43]–[45]. Investigating the GFP fluorescence emanating from these strains revealed a similar transient elevation in protein production during the fifth hour of sporulation (Figure 4). Thus, the increased translational activity observed within the forespore five hours into sporulation is not restricted to the ribosomal complex but is manifested by additional translational components. This coordinated expression seems to be a regulated process intended to equip the spore with sufficient translational capacity for completing its maturation and for future germination.

It is generally assumed that expression of translational machinery components is under the control of the housekeeping sigma factor A (σ^A^) [46]. However, the transient upregulation observed at a specific time point during sporulation may be due to activation of a forespore specific sigma factor. The most likely candidate to facilitate this increase is the forespore sigma factor G (σ^G^), which is active at the fifth hour of sporulation [47]–[49]. Further, σ^G^ was shown to contribute to the activation of σ^A^ transcription during sporulation [50], and thereby may indirectly activate the expression of transitional components. Indeed, inactivating σ^G^ reduced the expression of both RplA and the various *rrn* reporters within the forespore (Figure 4; Figure S5), implying that it enhances expression of the investigated components.

### Dynamics of the Translational Components during Spore Germination

**Figure 5:**
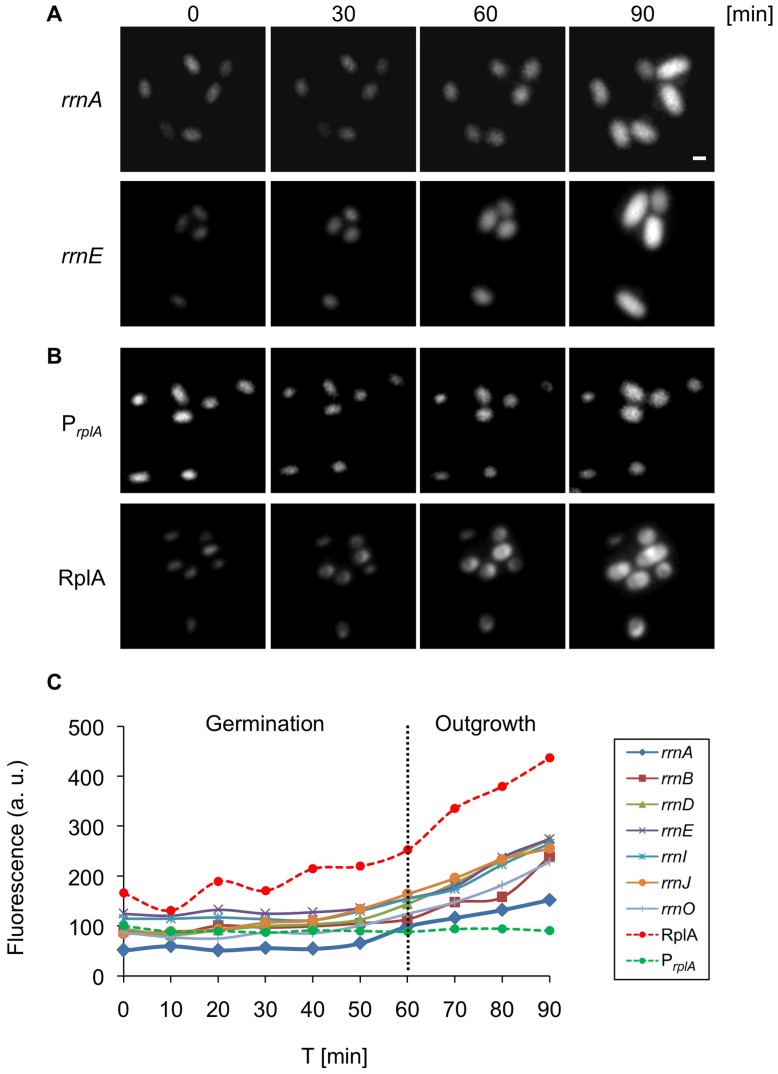
Expression of translational machinery components is biphasic during germination and outgrowth. (**A–B**) Strains carrying *P_rrnA_-gfp* (AR13) (A-*rrnA*), *P_rrnE_-gfp* (AR16) (A-*rrnE*), *P_rplA_-gfp* (AR25) (B-*P_rplA_*), *rplA-gfp* (AR5) (B-RplA) were germinated in rich LB medium and tracked by time lapse fluorescence microscopy. GFP fluorescence images acquired at the indicated time points [min] are shown. Of note, the localization of RplA-GFP changed during germination from a diffuse dispersion pattern (0 min) into a distinct focus (30 min). Scale bar corresponds to 1 µm. (**C**) Spores of strains carrying *P_rrn_-gfp* (*rrnO, A, B, D, E, I, J*), *P_rplA_-gfp* or *rplA-gfp* were germinated in rich LB medium and tracked by time lapse fluorescence microscopy. GFP fluorescence images were acquired and analyzed at the indicated time points [min]. The data is representative of one out of three independent biological repeats. Fluorescence from at least 50 cells was measured and averaged for each time point and is shown in arbitrary units (a.u.) (see Materials and Methods). Scale bar corresponds to 1 µm.

The processes of spore germination and outgrowth provide a unique opportunity to investigate the activity of the translational machinery during the switch from dormancy to a vegetative life form. To study this revival process at a single cell level, spores carrying the various *rrn* reporters were induced to germinate and followed by time lapse microscopy at 10 min intervals. During the first 50 min of germination, the fluorescence from all tested *rrn* promoters remained almost constant (Figure 5A and 5C). This could result from a steady state in *rrn* activity, or it may emanate from GFP molecules preserved within the spore and persist throughout this period. At the following time points, upon initiation of spore outgrowth, a gradual increase in *rrn* expression was detected prior to the first cell division. On the other hand, the activity of the *rplA* promoter was constant without any detectable increase during outgrowth, though the RplA protein, similarly to the *rrn* promoters, displayed increasing expression (Figure 5B and 5C). These results suggest that the spore probably encases sufficient levels of translational components enabling it to proceed through germination. However, the markedly increased expression of translational components is required to carry on outgrowth and cell division. This *rrn* expression pattern corresponds to the cellular rRNA levels measured previously during germination and outgrowth [31].

### The Relative Contribution of Each *Rrn* Promoter throughout the *B. Subtilis* Life Cycle

Monitoring the activity of the *rrn* promoters during the different phases of the *B. subtilis* life cycle exhibited diverse levels. However, under the majority of examined conditions, namely logarithmic phase, sporulation and germination, the activity profiles of the various *rrn* promoters were similar. According to our data (Figures 1, 3 and 5) the *rrn* operons can be classified into three groups based on expression strength: high - *rrnE*, medium - *rrnB,D,I,J,O* and low - *rrnA*. These results differ slightly from those described previously for exponential phase, whereby the activity of several *rrn* promoters was assessed using LacZ reporter fusions [42]. Consistent with our data, the strongest *rrn* promoter activity was associated with the *rrnE* operon that harbors three binding elements to the housekeeping sigma factor σ^A^, while the other members contain two such elements (Fig. S1B) [33], [51].

**Figure 6:**
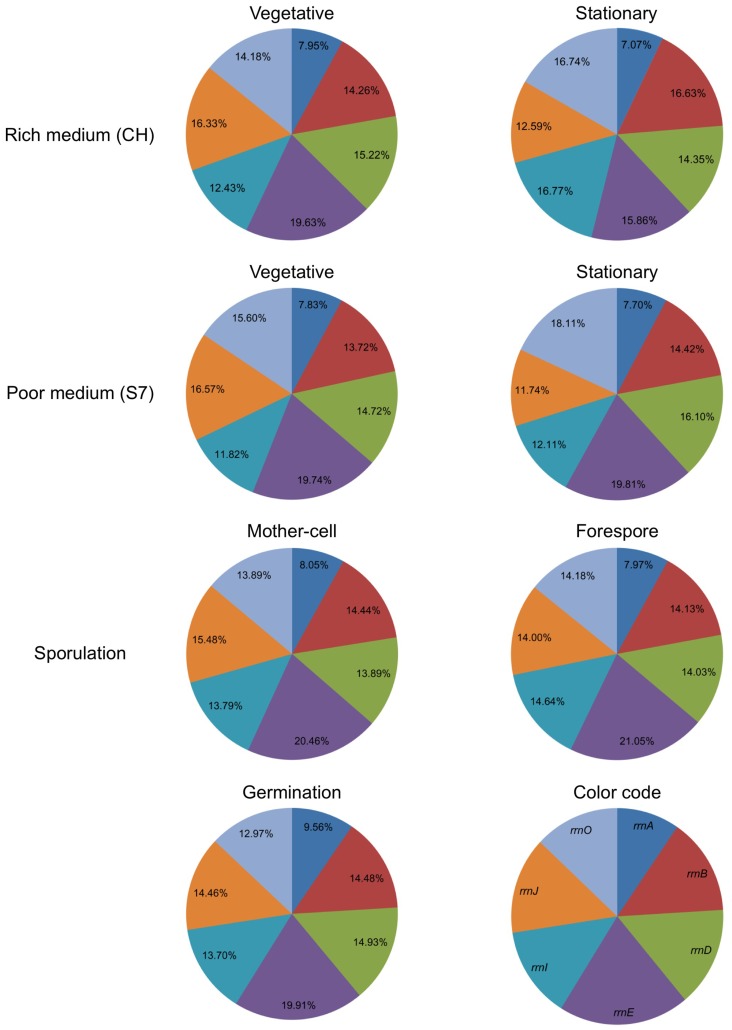
Contribution of each rrn promoter to overall translational activity. The contribution of each *rrn* promoter during different phases of *B. subtilis* life cycle is represented as an average percentage, with the sum activity of the seven tested promoters taken as 100%.

To determine the average contribution of each *rrn* operon to overall rRNA production during growth and development, we defined the sum activity of the seven tested promoters as 100% and calculated the proportional expression of a given promoter. The relative contribution of each promoter remained almost constant during vegetative growth (rich and poor medium), sporulation and germination (Figure 6). Hence, although the activity of each promoter is modulated throughout the bacterium life cycle, yet its proportional contribution remains the same. However, the contribution of some promoters changed during deep stationary phase both in rich and poor medium. In particular, the contributions of the *rrnB*,*D,I*,*O* operons, all displaying medium strength, became more prominent in poor medium at the expense of the remaining three promoters. We conclude that the relative contribution of *rrn* promoters to rRNA production is mainly constant but prone to modulation by external conditions particularly during deep stationary phase.

## Discussion

Proper activity and regulation of the translational machinery and the ability to modify and correspond to actual cellular demands, is crucial for all cell types. Particularly in bacteria, the capability to respond and regulate the expression of translation constituents is essential to adapt to fluctuating environments and out compete other species for limited resources. In this study, we have comprehensively monitored the expression of *B. subtilis* ribosomal components through the detection of the activity of *rrn* promoters and of RplA, representing the RNA and the proteinaceous components of the ribosome, respectively. Consistent with previous knowledge, we found the translational activity to be growth rate dependent in rich medium. However, when cells were grown in poor medium translational activity was relatively constant. Further, single cell analysis revealed the variability pattern of translational activity to dynamically vary among the cells, being influenced by nutrient availability. We have monitored for the first time the expression of ribosomal components during the developmental process of sporulation within the different cellular compartments of forespore and mother-cell. Our analysis uncovered an unexpected forespore-specific, transient increase in translational activity at a late stage of the process. This burst of expression is probably required to equip the spore with translational components needed for sporulation completion and germination. Finally, by evaluating the portion of each *rrn* contribution to the total *rrn* activity, we found the values for a given promoter to be relatively constant throughout the life cycle of the bacterium. Thus, tuning the expression of the *rrn* promoters in response to environmental cues requires a synchronized regulation.

Investigation of the differences between the *B. subtilis rrn* operons revealed relatively low variability rate in the rRNA coding sequence [26], suggesting that they may not differ in their function but mainly in their expression potential. The high copy number of *rrn* operons may represent a strategy to increase the range of translational activities that can be utilized by the bacterium. In this way, the bacterium can constantly monitor the environment and fine tune translational activity accordingly. The co-regulation that we described here may provide a strategy to rapidly achieve the optimal translational activity for a given condition state.

*B. subtilis rrn* promoters have been shown to be regulated by the housekeeping σ^A^ [46], [52], [53]. We observed an additional level of regulation in the *rrn* expression that is mediated by the forespore specific σ^G^. This complex regulation, employed during differentiation, may be exerted through the ability of σ^G^ to enhance σ^A^ transcription and in turn elevate the *rrn* operons activity. Consistently, it has been reported that two out of seven σ^A^ promoter elements are active only at late stages of sporulation, with one of them shown to be dependent on σ^G^ [50], [54]. The increase in the additional translational components detected here, at the time of σ^G^ activation, suggests the elevated expression of housekeeping genes to be a general phenomenon restricted not only to the ribosomal constituents. The wide rise in translational activity can be also facilitated by delivery of metabolites from the mother-cell to the forespore. This exchange may be conducted via the feeding tube formed during this stage connecting both compartments and affecting the forespore metabolic state [55]–[57].

Although we have found that the *rrn* promoters are for the most part coordinately regulated, four *rrn* promoters *rrnB,D,I,O* were distinctively upregulated during stationary phase in poor medium. In light of this finding, differential regulation of *rrn* operons is a plausible mechanism under specific conditions. In support of this view, it has been shown that one of the three *rrn* operons in the bacterium *Haloarcula marismortui* is specifically induced at high temperatures and its deletion leads to a temperature sensitive growth phenotype [58]. Similarly, the six *rrn* operons of *Streptomyces coelicolor* are differentially expressed during its morphological development [59], [60]. Notably, pronounced differences are evident across the *rrn* coding sequences in these organisms, raising the possibility that ribosomes with alternative features are formed. It remains to be resolved whether unique role is assigned to each *rrn* operon in *B. subtilis*, and if so whether ribosomes with differential structure or/and function exist. In this view, it has been shown that different types of ribosomes are being produced in *B. subtilis* in accordance with zinc availability [61]–[63].

*rrn* transcription has been previously shown to be regulated by the levels of initiating nucleoside triphosphate (iNTP) and (p)ppGpp [1]. In turn, the concentration of these molecules is responsive to amino acids starvation as part of the stress induced stringent response [64]. We observed a significant down regulation of *rrn* transcription when the cells entered stationary phase in rich medium and when sporulation was triggered, conditions known to activate the stringent response [65]–[67]. On the other hand, no such reduction in expression was observed when cells entered stationary phase in poor medium, suggesting the transition to be controlled by additional pathways.

Monitoring the *rrn* transcriptional variability among individual cells revealed detectable increase when cells were grown in poor medium and during stationary phase. Elevating variability under challenging conditions may represent a strategy to enhance population survival. Indeed, it has been suggested that increase in variability has a positive effect on survival rates in unpredictable environments [68]–[70]. Furthermore, transcriptional variability among different bacterial cells can lead to the emergence of subpopulations with increased resistance to antibiotics [71]. In this respect, we have previously demonstrated that transcriptional and translational errors are elevated under challenging conditions, thereby increasing population diversity [72]. Variability in translational activity among single cells monitored here may represent an additional strategy to enhance population robustness.

## Materials and Methods

### Strains and General Methods

Plasmids and primers used for this study are described in Table S1 and Table S2, respectively. *B. subtilis* strains were derivatives of the wild type strain PY79 [73] and are listed in Table S3. All general methods were performed as described previously [74]. For vegetative growth experiments *B. subtilis* cells were grown in hydrolyzed casein (CH) or S7 [75] minimal medium at 37°C. Sporulation was induced by resuspension in Sterlini and Mandelstam medium [74], [76] of cultures grown in (CH) medium to an OD_600_ of 0.5. Germination experiments were carried out in LB medium [77]. For all experiments, cultures were inoculated to an OD_600_ of 0.05 using an overnight culture grown in the same medium.

### General Fluorescence Microscopy Methods

Fluorescence microscopy was carried out as described previously [72]. For all experiments, cells were visualized and photographed using an Axio Observer Z1 microscope (Zeiss) equipped with a CoolSnap HQII camera (Photometrics; Roper Scientific) and X-cite (120PC; EXFO) as a fluorescence illumination light source. System control and image processing were performed using MetaMorph software (version 7.5; Molecular Devices). For sporulation, samples (0.5 ml) of culture were removed, gently centrifuged and resuspended in 10 µl of PBS ×1 (Phosphate-Buffered Saline) supplemented with fluorescent membrane stain FM4–64 (Molecular Probes, Eugene, OR) at 1 µg/ml. For time-lapse microscopy observations, a mounting frame (A-7816, Invitrogen) was filled with 1% LB agarose. Spores were purified by repetitive washes with sterile DDW, and samples (0.1 ml) were removed, centrifuged briefly, and spotted on the agarose pad. Spores were incubated and germinated at 37°C in a temperature controlled chamber (Pecon-Zeiss, Germany) tracked, visualized and photographed with the system described above.

### Fluorescent Image Processing

Initially, the average fluorescence intensity of individual cells was determined. For growth and germination, cell boundaries were defined by the phase contrast image and the average fluorescence emanating from each cell was calculated from the corresponding fluorescence image. In sporulation, at the initial time points (0–1hrs), before asymmetric division, cell boundaries were defined as described above. In 2–3 hrs of sporulation the mother-cell and forespore compartments were identified using membrane stain FM4–64. During the following time points (4–9 hrs) mother-cell, forespore and mature spores edges were determined by phase contrast images. The average background fluorescence signal was calculated from cell-free regions within a given field. The background level was subtracted from the fluorescent values obtained for each individual cell. The fluorescence intensity of a population of cells within a given field was determined by averaging the fluorescence intensity from all individual cells. To finally determine the average GFP signal emanating from a given strain shown in arbitrary units (a.u.), the calculated net average fluorescence intensity from at least 3 different fields was averaged and the intensity of a wild type (PY79) strain lacking the GFP gene subtracted. Coefficient of Variation (CV) of fluorescence level was calculated as the Standard Deviation (SD) divided by the average fluorescence for each time point.

## Supporting Information

Figure S1 **Characterization of the array of strains used in this study.** (**A**) Location of the *rrn* operons and the *rplA* gene on the *B*. *subtilis* chromosome. (**B**) A schematic representation of the P*_rrn_*-*gfp* fusions at *amyE* locus. The cloned promoter fragments, shown in blue and confined by the dashed lines, contain the intergenic region preceding each indicated *rrn* operon. Arrows designate *bona fide* promoter sequences. Promoter regions were amplified using primers listed in Table S2. (**C**) Growth curves of wild type (PY79) and *P_rrn_-gfp* (*rrnO, A, B, D, E, I, J*), *P_rplA_-gfp* or *rplA-gfp* strains grown in rich medium (CH) or in minimal medium (S7).
(TIF)

Figure S2 **Half-life determination of GFPmut2 in** ***B.*** ** ***subtilis*** **.** Stability of GFPmut2 was monitored in *B. subtilis* cells (SB444) harboring *P_hyperspank_-gfp*. Cells were grown in minimal medium (S7) containing the inducer till OD_600_ 1.0, and then were transferred to fresh medium in the absence of the inducer, and fluorescence was followed for 650 min (see Materials & Methods S1). The calculated GFP half-life is approximately 5.6 hrs as determined by using the equation T1/2 = −ln2/µ, were µ is the slope of the curve (the slope constant, µ, was determined to be approximately −0.123 min^−1^). Of note, GFPmut3 with half life of approximately 10 hrs was utilized successfully as a reporter for genome wide expression analyses in *B. subtilis* [34]–[36].
(TIF)

Figure S3 **Observing** ***rrn*** **promoter activity during deep stationary phase.** FRAP experiment of *P_rrnO_-gfp (*AR17) cells grown to a deep stationary phase in minimal medium (S7). At t0 cells were photobleached to reduce the GFP signal, photographed, and followed for their growth and fluorescence recovery at the indicated time intervals [min]. Upper panels show fluorescent images while lower panels show the corresponding phase contrast images. Fluorescence images have been normalized to the same intensity range. Scale bar corresponds to 1 µm.
(TIF)

Figure S4 **rRNA profile upon resuscitation from stationary phase.** (**A**) Bioanalyzer pseudogel of RNA extracted from equal number of wild type cells (PY79) grown in minimal medium (S7) over time (0.5–24 hrs). Next, deep stationary cells (24 hrs) were resuscitated in fresh poor medium and RNA was extracted from equal number of cells after 1 hr (1* hrs). Arrows designate the positions of 23 S rRNA (upper) and 16 S rRNA (lower). Quantification of 23 S and 16 S rRNA band intensities (a.u.) is presented below each lane (see Materials & Methods S1). All lanes in the pseudogel are scaled to the same intensity range. (**B**) Strains carrying *P_rrn_-gfp (rrnO, A, B, D, E, I, J), P_rplA_-gfp or rplA-gfp* were grown in poor medium (S7) for 24 hrs. Cells were then resuscitated in fresh poor medium (S7) and samples were taken at the indicated time points [hrs] and the GFP signal monitored using fluorescence microscopy. t0 represents the fluorescence intensity prior to resuscitation. Fluorescence from at least 100 cells was measured and averaged for each time point, and is shown in arbitrary units (a.u.) (see Materials and Methods). (**C**) Cells carrying PrrnO-gfp (AR17) were transferred from stationary conditions in minimal medium (S7), into rich medium (LB) and tracked by time lapse fluorescence microscopy. Shown are phase (upper panels) and GFP fluorescence images (lower panels) acquired at the indicated time points [min]. Dashed-line frames highlight cells displaying low rrn promoter activity at t0 that lyse during time. Scale bar corresponds to 1 µm.
(TIF)

Figure S5 **Inactivating sigma G reduces the expression of the various** ***rrn*** **reporters.** Strains carrying *P_rrnA_-gfp* (AR13), *spoIIIG::cat, P_rrnA_-gfp* (AR45), *P_rrnE_-gfp* (AR16) and *spoIIIG::cat, P_rrnE_-gfp* (AR48) were induced to sporulate and samples taken at the indicated time points [hrs]. Shown are phase contrast images (left panels) and corresponding GFP fluorescence images (right panels). Arrowheads designate the position of forespores. Scale bar corresponds to 1 µm.
(TIF)

Materials & Methods S1. (DOC)

Table S1 **List of plasmids.**
(DOC)

Table S2 **List of primers.**
(DOC)

Table S3 ***B. subtilis*** **strains used in this study.**
(DOC)
