# Supplementary figures and images for: Dynamic Expression of the Translational Machinery during Bacillus subtilis Life Cycle at a Single Cell Level
Source: PLoS One. 2012 Jul 25;7(7):e41921. doi: 10.1371/journal.pone.0041921 (PMC3405057; doi:10.1371/journal.pone.0041921)

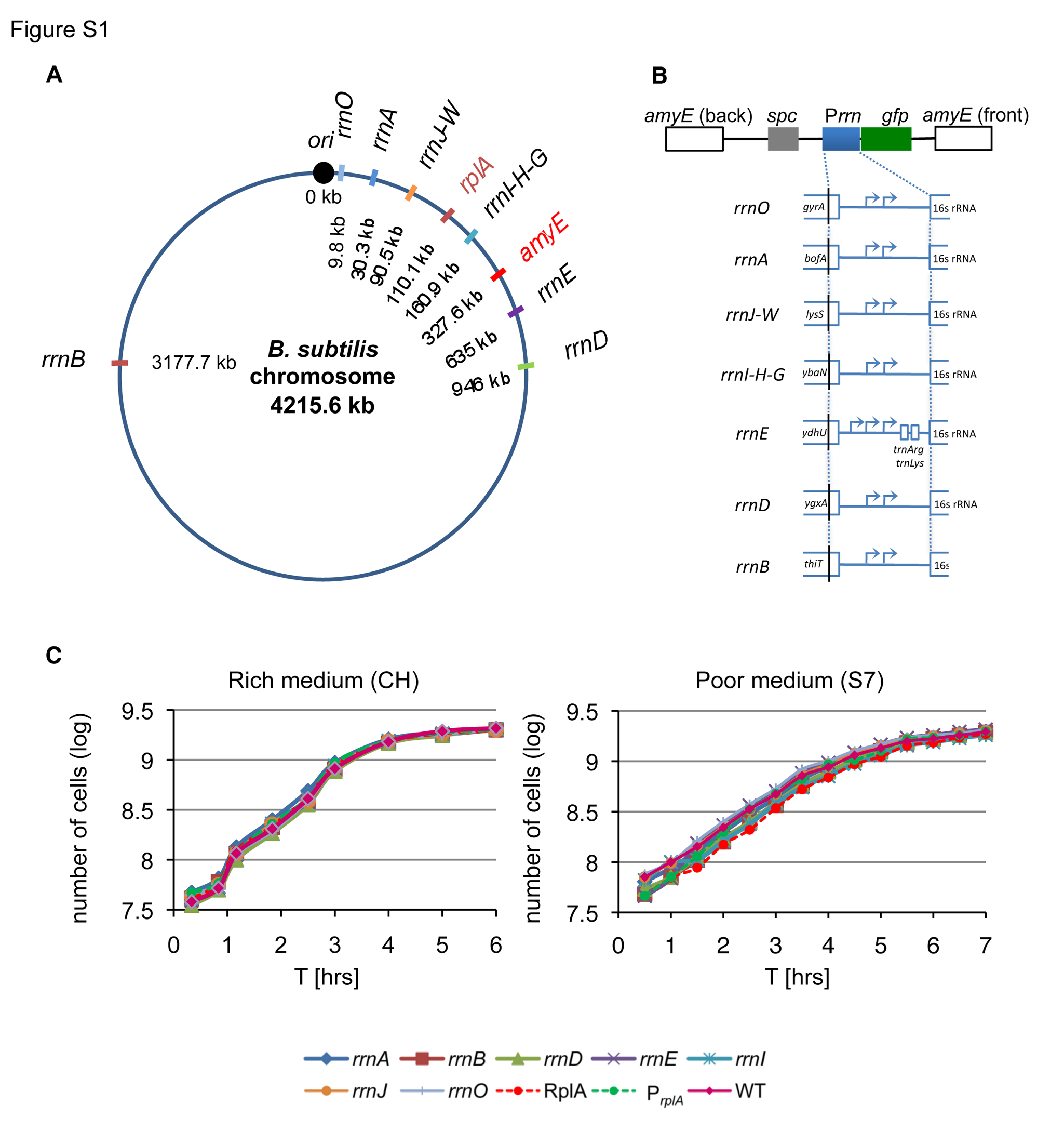

Supplement: Figure S1 — Characterization of the array of strains used in this study. (A) Location of the rrn operons and the rplA gene on the B. subtilis chromosome. (B) A schematic representation of the Prrn-gfp fusions at amyE locus. The cloned promoter fragments, shown in blue and confined by the dashed lines, contain the intergenic region preceding each indicated rrn operon. Arrows designate bona fide promoter sequences. Promoter regions were amplified using primers listed in Table S2. (C) Growth curves of wild type (PY79) and Prrn-gfp (rrnO, A, B, D, E, I, J), PrplA-gfp or rplA-gfp strains grown in rich medium (CH) or in minimal medium (S7). (TIF) [file pone.0041921.s001.tif]

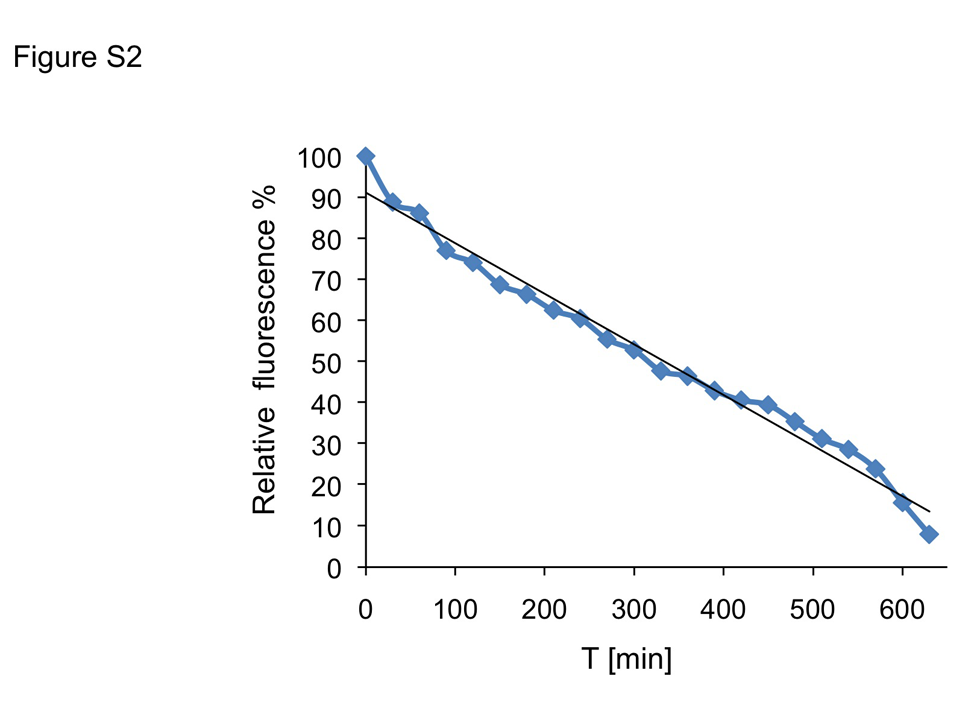

Supplement: Figure S2 — Half-life determination of GFPmut2 in B. subtilis . Stability of GFPmut2 was monitored in B. subtilis cells (SB444) harboring Phyperspank-gfp. Cells were grown in minimal medium (S7) containing the inducer till OD600 1.0, and then were transferred to fresh medium in the absence of the inducer, and fluorescence was followed for 650 min (see Materials & Methods S1). The calculated GFP half-life is approximately 5.6 hrs as determined by using the equation T1/2 = −ln2/µ, were µ is the slope of the curve (the slope constant, µ, was determined to be approximately −0.123 min−1). Of note, GFPmut3 with half life of approximately 10 hrs was utilized successfully as a reporter for genome wide expression analyses in B. subtilis [34]–[36]. (TIF) [file pone.0041921.s002.tif]

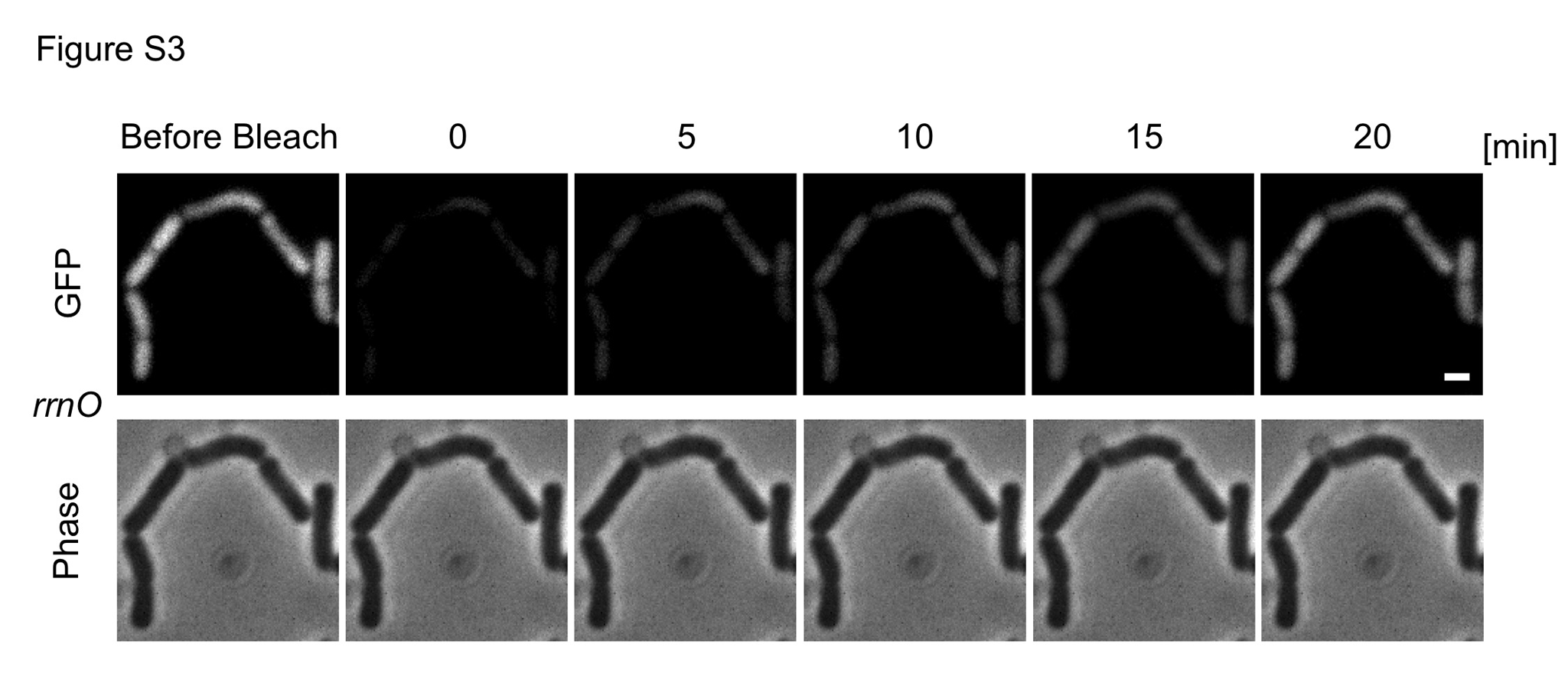

Supplement: Figure S3 — Observing rrn promoter activity during deep stationary phase. FRAP experiment of PrrnO-gfp (AR17) cells grown to a deep stationary phase in minimal medium (S7). At t0 cells were photobleached to reduce the GFP signal, photographed, and followed for their growth and fluorescence recovery at the indicated time intervals [min]. Upper panels show fluorescent images while lower panels show the corresponding phase contrast images. Fluorescence images have been normalized to the same intensity range. Scale bar corresponds to 1 µm. (TIF) [file pone.0041921.s003.tif]

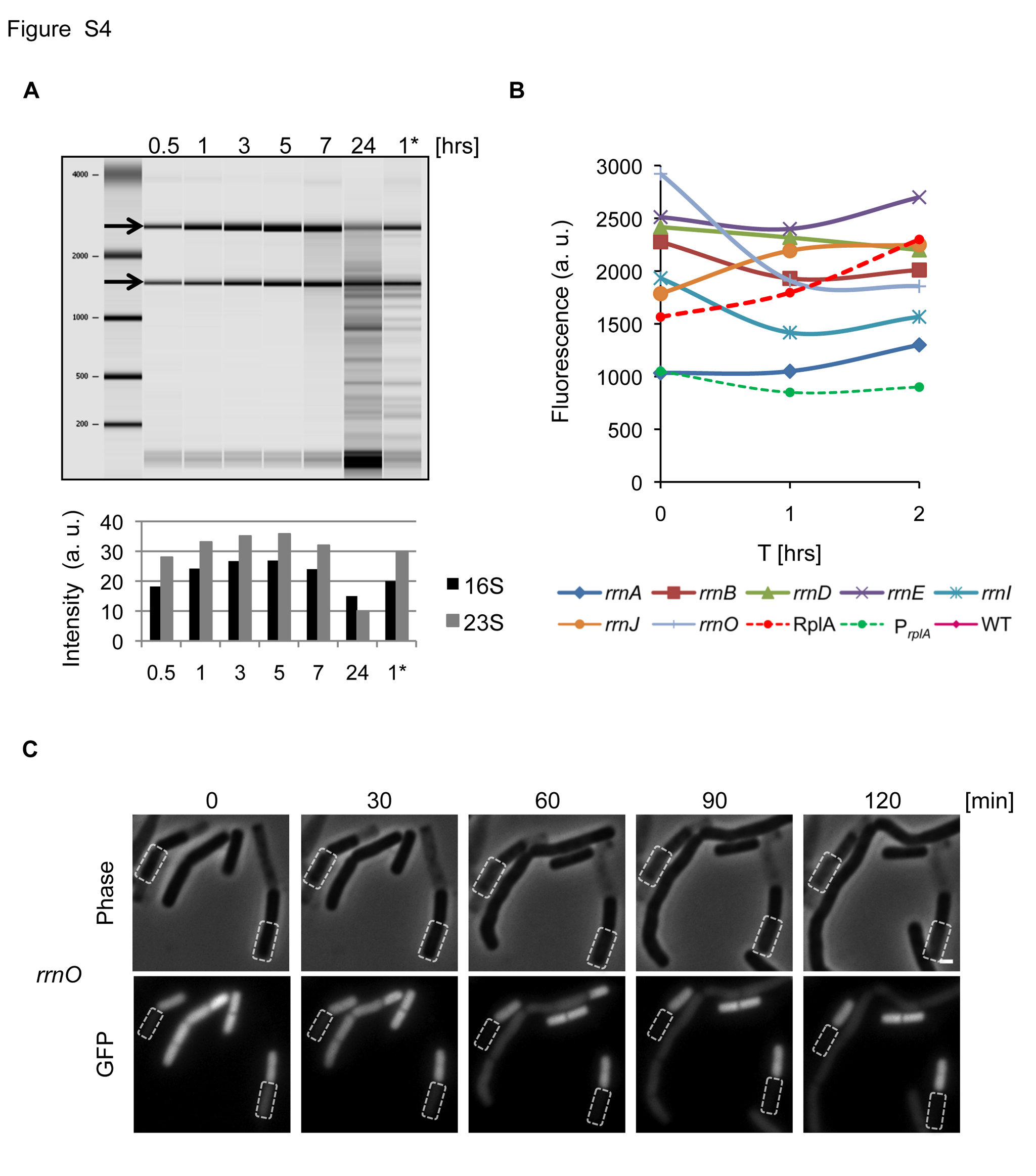

Supplement: Figure S4 — rRNA profile upon resuscitation from stationary phase. (A) Bioanalyzer pseudogel of RNA extracted from equal number of wild type cells (PY79) grown in minimal medium (S7) over time (0.5–24 hrs). Next, deep stationary cells (24 hrs) were resuscitated in fresh poor medium and RNA was extracted from equal number of cells after 1 hr (1* hrs). Arrows designate the positions of 23 S rRNA (upper) and 16 S rRNA (lower). Quantification of 23 S and 16 S rRNA band intensities (a.u.) is presented below each lane (see Materials & Methods S1). All lanes in the pseudogel are scaled to the same intensity range. (B) Strains carrying Prrn-gfp (rrnO, A, B, D, E, I, J), PrplA-gfp or rplA-gfp were grown in poor medium (S7) for 24 hrs. Cells were then resuscitated in fresh poor medium (S7) and samples were taken at the indicated time points [hrs] and the GFP signal monitored using fluorescence microscopy. t0 represents the fluorescence intensity prior to resuscitation. Fluorescence from at least 100 cells was measured and averaged for each time point, and is shown in arbitrary units (a.u.) (see Materials and Methods). (C) Cells carrying PrrnO-gfp (AR17) were transferred from stationary conditions in minimal medium (S7), into rich medium (LB) and tracked by time lapse fluorescence microscopy. Shown are phase (upper panels) and GFP fluorescence images (lower panels) acquired at the indicated time points [min]. Dashed-line frames highlight cells displaying low rrn promoter activity at t0 that lyse during time. Scale bar corresponds to 1 µm. (TIF) [file pone.0041921.s004.tif]

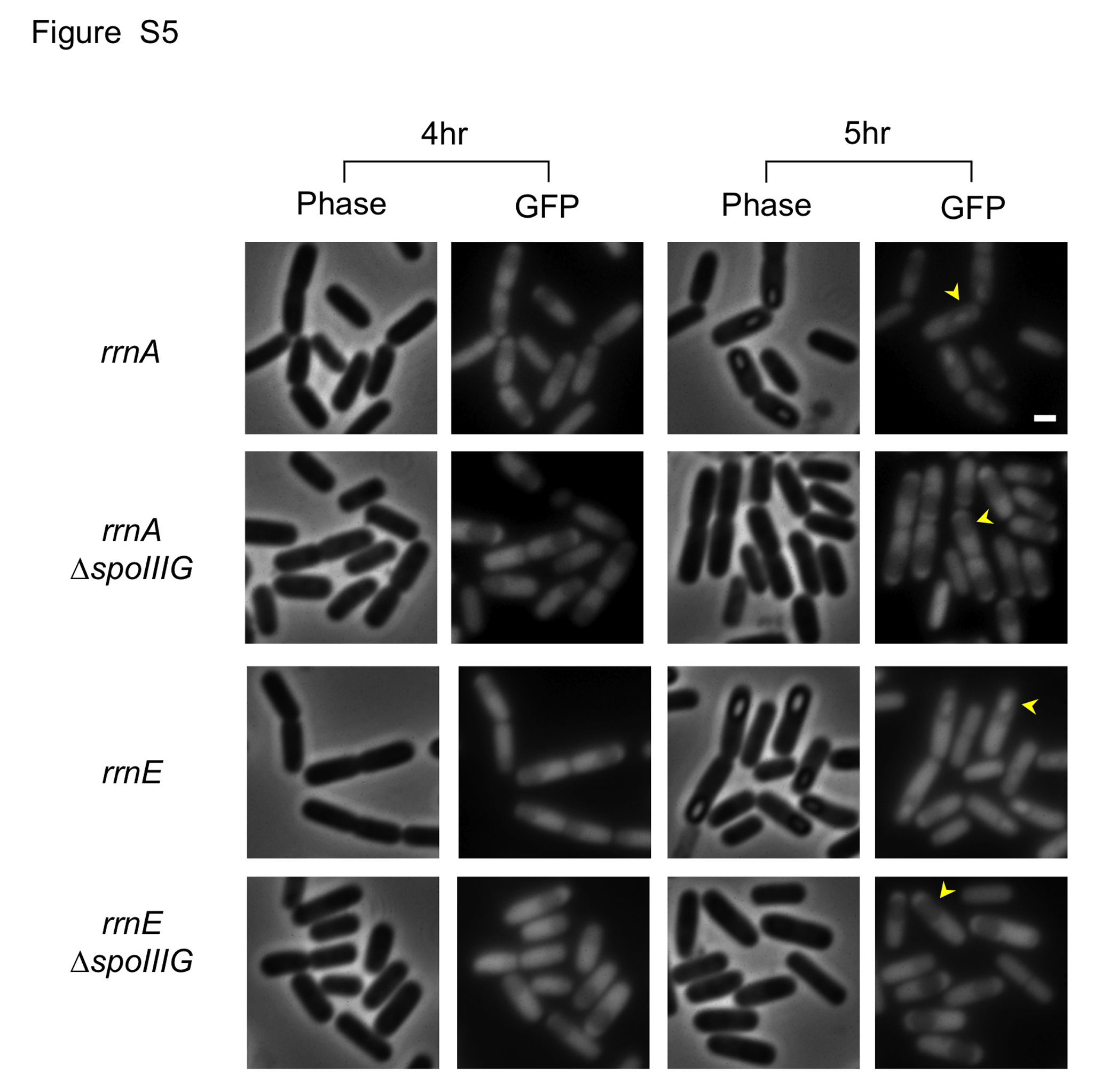

Supplement: Figure S5 — Inactivating sigma G reduces the expression of the various rrn reporters. Strains carrying PrrnA-gfp (AR13), spoIIIG::cat, PrrnA-gfp (AR45), PrrnE-gfp (AR16) and spoIIIG::cat, PrrnE-gfp (AR48) were induced to sporulate and samples taken at the indicated time points [hrs]. Shown are phase contrast images (left panels) and corresponding GFP fluorescence images (right panels). Arrowheads designate the position of forespores. Scale bar corresponds to 1 µm. (TIF) [file pone.0041921.s005.tif]
