# Supplementary material for: Dynamic Expression of the Translational Machinery during Bacillus subtilis Life Cycle at a Single Cell Level
Source: PLoS One. 2012 Jul 25;7(7):e41921. doi: 10.1371/journal.pone.0041921 (PMC3405057; doi:10.1371/journal.pone.0041921)
Supplement: Materials & Methods S1. — (DOC) [file pone.0041921.s006.doc]

**GFP half-life determination**

The determination of GFP half-life was done as described previously [1] with several modifications. Briefly, a strain SB444 harboring the *P_hyperspank_-gfp* gene were grown exponentially in S7 minimal medium supplemented with 0.5 mM IPTG at 37°C. At an OD_600_ 1.0 the cultures were harvested, washed in S7 minimal medium without IPTG, shifted to the same volume of preheated (37°C) S7 medium and divided into a 12- well plate at a final volume of 1ml culture per well. The plates were covered with an adhesive pad (Breathe-Easy™ sealing membrane, Sigma) to prevent evaporation and grown in a Wallac Victor2 multiwell fluorimeter at 37°C, set with constant shaking (3 mm orbital, fast speed). The fluorescence (excitation 485nm, emission 535nm, 0.1 sec, CW lamp energy setting 8,256) was measured at the designated intervals. The measured fluorescence values of the culture were corrected for background fluorescence by subtracting the corresponding measured fluorescence values of SB444, which was grown in parallel without the addition of IPTG at any stage. Finally, the background corrected values of fluorescence were converted into relative green fluorescence and plotted as a function of post-shift time (relative fluorescence was arbitrarily set to 100% in t0 samples). GFP half-life was determined by using the equation T1/2=−ln2/μ, were μ is the slope of the curve.

**Fluorescence Recovery After Photobleaching (FRAP)**

For FRAP experiments, strains were grown for 24 hrs in S7 medium to deep stationary phase. Samples of 0.1ml were concentrated, applied to an agarose pad composed of supernatant of the same culture and incubated at 37°C in a temperature controlled chamber (PeCon). At t0 cells were photobleached to reduce the GFP signal, photographed at different time intervals, and followed for their growth and fluorescence recovery.

**RNA extraction and analysis**

The rRNA level and pattern during growth up until deep stationary phase and upon resuscitation was carried out as follows: RNA was extracted from the same amount of cells (~1.2x10^10^) using fast RNA Pro Blue kit (MP Biomedicals) with the following modifications: *B. subtilis* cultures were processed three times with the FastPrep-24 (MP Biomedicals) in setting 6.5 m/s, for 45 s each time, with 5 min of ice cooling between cycles. RNA samples were visualized by capillary gel electrophoresis in the Agilent 2100 Bioanalyzer. Band intensities were quantified by comparing the total intensity of identical sized regions using MetaMorph 7.5 software (Molecular Devices).

**References**

1. Andersen JB, Sternberg C, Poulsen LK, Bjorn SP, Givskov M, et al. (1998) New unstable variants of green fluorescent protein for studies of transient gene expression in bacteria. Appl Environ Microbiol 64: 2240-2246.
