# Supplementary material for: Dynamic Expression of the Translational Machinery during Bacillus subtilis Life Cycle at a Single Cell Level
Source: PLoS One. 2012 Jul 25;7(7):e41921. doi: 10.1371/journal.pone.0041921 (PMC3405057; doi:10.1371/journal.pone.0041921)
Supplement: Table S1 — List of plasmids. (DOC) [file pone.0041921.s007.doc]

**Table S1. List of plasmids**

| **Plasmid** | **Genotype** | **Description** |
| --- | --- | --- |
| pDR111 | *amyE::* P*_hyper-spank_ -spc* | A gift from David Rudner, HMS |
| pKL147 | *spc-gfpmut2* | [1] |
| pAR4 | *rplA-gfpmut2* | Constructed by amplifying the 3’ region of *rplA* gene by PCR using the primers 501 and 502 (Table S2), which replaced the stop codon with *Xho*I site. The PCR-amplified DNA was digested with *EcoRI* and *Xho*I and was cloned into the *Eco*RI and *Xho*I sites of pKL147 (*spc*). |
| pAR8 | *thrS-gfpmut2* | Constructed by amplifying the 3’ region of *thrS* gene by PCR using the primers 690 and 691 (Table S2), which replaced the stop codon with *Xho*I site. The PCR-amplified DNA was digested with *EcoRI* and *Xho*I and was cloned into the *Eco*RI and *Xho*I sites of pKL147 (*spc*). |
| pAR9 | *rnpA-gfpmut2* | Constructed by amplifying the 3’ region of *rnpA* gene by PCR using the primers 692 and 693 (Table S2), which replaced the stop codon with *Xho*I site. The PCR-amplified DNA was digested with *EcoRI* and *Xho*I and was cloned into the *Eco*RI and *Xho*I sites of pKL147 (*spc*). |
| pAR10 | *gltX-gfpmut2* | Constructed by amplifying the 3’ region of *gltX*  gene by PCR using the primers 694 and 695 (Table S2), which replaced the stop codon with *Xho*I site. The PCR-amplified DNA was digested with *EcoRI* and *Xho*I and was cloned into the *Eco*RI and *Xho*I sites of pKL147 (*spc*). |
| pAR13 |  | Constructed by amplifying the *gfpmut2* gene from pKL147, by PCR using primers 807 and 50 (Table S2). The PCR-amplified DNA was digested with *SphI* and *EcoRI* and was cloned into pDR111 digested with the same enzymes. |
| pAR16 | *amyE::P_rrnA_-gfpmut2-spc* | Constructed by amplifying the *rrnA* promoter, by PCR using primers 809 and 816 (Table S2). The PCR-amplified DNA was digested with *BamHI* and *SphI* and was cloned into pAR13 digested with the same enzymes. |
| pAR17 | *amyE::P_rrnB_-gfpmut2-spc* | Constructed by amplifying the *rrnB* promoter, by PCR using primers 814 and 815 (Table S2). The PCR-amplified DNA was digested with *BamHI* and *SphI* and was cloned into pAR13 digested with the same enzymes. |
| pAR18 | *amyE::P_rrnD_-gfpmut2-spc* | Constructed by amplifying the *rrnD* promoter, by PCR using primers 813 and 815 (Table S2). The PCR-amplified DNA was digested with *BamHI* and *SphI* and was cloned into pAR13 digested with the same enzymes. |
| pAR19 | *amyE::P_rrnE_-gfpmut2-spc* | Constructed by amplifying the *rrnE* promoter, by PCR using primers 812 and 815 (Table S2). The PCR-amplified DNA was digested with *BamHI* and *SphI* and was cloned into pAR13 digested with the same enzymes. |
| pAR20 | *amyE::P_rrnI_-gfpmut2-spc* | Constructed by amplifying the *rrnI* promoter, by PCR using primers 915 and 816 (Table S2). The PCR-amplified DNA was digested with *BamHI* and *SphI* and was cloned into pAR13 digested with the same enzymes. |
| pAR21 | *amyE::P_rrnJ_-gfpmut2-spc* | Constructed by amplifying the *rrnJ* promoter, by PCR using primers 810 and 816 (Table S2). The PCR-amplified DNA was digested with *BamHI* and *SphI* and was cloned into pAR13 digested with the same enzymes. |
| pAR22 | *amyE::P_rrnO_-gfpmut2-spc* | Constructed by amplifying the *rrnO* promoter, by PCR using primers 808 and 816 (Table S2). The PCR-amplified DNA was digested with *BamHI* and *SphI* and was cloned into pAR13 digested with the same enzymes. |
| pAR26 | *amyE::P_rplA_-gfpmut2-spc* | Constructed by amplifying the promoter region of the *rplK-rplA* operon, by PCR using primers 1032 and 1033 (Table S2). The PCR-amplified DNA was digested with *BamHI* and *SphI* and was cloned into pAR13 digested with the same enzymes. |

**References**

1. Lemon KP, Grossman AD (1998) Localization of bacterial DNA polymerase: evidence for a factory model of replication. Science 282: 1516-1519.
