# Supplementary material for: Dynamic Expression of the Translational Machinery during Bacillus subtilis Life Cycle at a Single Cell Level
Source: PLoS One. 2012 Jul 25;7(7):e41921. doi: 10.1371/journal.pone.0041921 (PMC3405057; doi:10.1371/journal.pone.0041921)
Supplement: Table S2 — List of primers. (DOC) [file pone.0041921.s008.doc]

**Table S2. List of primers**

| **Primer name** | **Primer sequence (5’-3’)** |
| --- | --- |
| 050 | ACCTAGGAATTCTTATTTGTATAGTTCATCCATGC |
| 501 | TGGATCGAATTCAGCTGCTAAGCTTGTAGACCATTC |
| 502 | ACCTAGCTCGAGTTTTACGTTAAAAGTTGAAGAGTCTAC |
| 690 | TGGATCGAATTCCTAGGCAGCAAAGAGCTGTATG |
| 691 | TCGATCCTCGAGTTTTTTTCGCTTCAGCTACTGCC |
| 692 | TGGATCGAATTCTTGAAGAAGCGAAATCG |
| 693 | TCGATCCTCGAGCTTTGACGAAGATTTC |
| 694 | TGGATCGAATTCGGACGGAACGCCTACTTATAAC |
| 695 | TCGATCCTCGAGGATATTCTTTAAACGCTGAATTGCAGTCTC |
| 807 | TGCGCATGCTAAGGAGGAAATTTTAAAATGAGTAAAGGAGAAGAACTTTTC |
| 808 | TCTGGATCCAGAATGGACATCAATGATATCTCC |
| 809 | AACCGGATCCGGTTACAACAGCTATCAGCG |
| 810 | TTCGGGATCCCGAATGCCCCTTCTATTCGC |
| 812 | CTTCGGATCCGAAGCAGGTTATCCAGCAGG |
| 813 | TCCCGGATCCGGGACTTATCAACGTCTGCC |
| 814 | GCCGGGATCCCGGCTGCCGCTGTCATAATG |
| 815 | CCAGCATGCTGGATCACAGGTTAAGTTCACCGC |
| 816 | TACGCATGCGTAGTTTGACTGACTACGCAC |
| 915 | TTCGGGATCCAGTATCGGTTCTGCTCTCTCATCACCC |
| 1032 | ACCTAGGGATCCTGTGAAAAAACTTGAAATTGCTAT |
| 1033 | ACCTAGAAGCTTGAGACACACCTCCTTAAGT |
