# Supplementary material for: Dynamic Expression of the Translational Machinery during Bacillus subtilis Life Cycle at a Single Cell Level
Source: PLoS One. 2012 Jul 25;7(7):e41921. doi: 10.1371/journal.pone.0041921 (PMC3405057; doi:10.1371/journal.pone.0041921)
Supplement: Table S3 — B. subtilis strains used in this study. (DOC) [file pone.0041921.s009.doc]

**Table S3. *B. subtilis*** strains used in this study

| **Strain** | **Genotype** | **Comments** |
| --- | --- | --- |
| PY79 | Wild type | [1] |
| RL560 | *spoIIIG::cat* | [2] |
| SB444 | *amyE::P_hyper-spank_-gfpmut2-spc* | [3] |
| AR5 | *rplA-gfpmut2-spc* | *rplA-gfpmut2-spc* allele was constructed using plasmid pAR5. |
| AR9 | *thrS-gfpmut2-spc* | *thrS-gfpmut2-spc* allele was constructed by transforming PY79 with plasmid pAR8. |
| AR10 | *rnpA-gfpmut2-spc* | *rnpA-gfpmut2-spc* allele was constructed by transforming PY79 with plasmid pAR9. |
| AR11 | *gltX-gfpmut2-spc* | *gltX-gfpmut2-spc* allele was constructed by transforming PY79 with plasmid pAR10. |
| AR13 | *amyE::P_rrnA_-gfpmut2-spc* | *amyE::P_rrnA_-gfpmut2-cat* allele was constructed by transforming PY79 with plasmid pAR16. |
| AR14 | *amyE::P_rrnB_-gfpmut2-spc* | *amyE::P_rrnB_-gfpmut2-spc* allele was constructed by transforming PY79 with plasmid pAR17. |
| AR15 | *amyE::P_rrnD_-gfpmut2-spc* | *amyE::P_rrnD_-gfpmut2-spc* allele was constructed by transforming PY79 with plasmid pAR18. |
| AR16 | *amyE::P_rrnE_-gfpmut2-spc* | *amyE::P_rrnE_-gfpmut2-spc* allele was constructed by transforming PY79 with plasmid pAR19. |
| AR17 | *amyE::P_rrnO_-gfpmut2-spc* | *amyE::P_rrnO_-gfpmut2-spc* allele was constructed by transforming PY79 with plasmid pAR22. |
| AR18 | *amyE::P_rrnI_-gfpmut2-spc* | *amyE::P_rrnI_-gfpmut2-spc* allele was constructed by transforming PY79 with plasmid pAR20. |
| AR19 | *amyE::P_rrnJ_-gfpmut2-spc* | *amyE::P_rrnJ_-gfpmut2-spc* allele was constructed by transforming PY79 with plasmid pAR21. |
| AR20 | *spoIIIG::cat, rplA-gfpmut2-spc* | Constructed by transforming AR5 strain with the genomic DNA from RL560. |
| AR25 | *amyE::P_rplA_-gfpmut2-spc* | *amyE::P_rplA_-gfpmut2-spc* allele was constructed by transforming PY79 with plasmid pAR26. |
| AR45 | *spoIIIG::cat, amyE::P_rrnA_-gfpmut2-spc* | Constructed by transforming AR13 strain with the genomic DNA from RL560. |
| AR46 | *spoIIIG::cat, amyE::P_rrnB_-gfpmut2-spc* | *C*onstructed by transforming AR14 strain with the genomic DNA from RL560. |
| AR47 | *spoIIIG::cat, amyE::P_rrnD_-gfpmut2-spc* | Constructed by transforming AR15 strain with the genomic DNA from RL560. |
| AR48 | *spoIIIG::cat, amyE::P_rrnE_-gfpmut2- spc* | Constructed by transforming AR16 strain with the genomic DNA from RL560. |
| AR49 | *spoIIIG::cat, amyE::P_rrnI_-gfpmut2-spc* | Constructed by transforming AR18 strain with the genomic DNA from RL560. |
| AR50 | *spoIIIG::cat, amyE::P_rrnJ_-gfpmut2-spc* | Constructed by transforming AR19 strain with the genomic DNA from RL560. |
| AR51 | *spoIIIG::cat, amyE::P_rrnO_-gfpmut2-spc* | Constructed by transforming AR17 strain with the genomic DNA from RL560. |

**References**

1. Youngman P, Perkins JB, Losick R (1984) Construction of a cloning site near one end of Tn917 into which foreign DNA may be inserted without affecting transposition in *Bacillus subtilis* or expression of the transposon-borne *erm* gene. Plasmid 12: 1-9.

2. Cutting S, Roels S, Losick R (1991) Sporulation operon *spoIVF* and the characterization of mutations that uncouple mother-cell from forespore gene expression in *Bacillus subtilis*. J Mol Biol 221: 1237-1256.

3. Meyerovich M, Mamou G, Ben-Yehuda S (2010) Visualizing high error levels during gene expression in living bacterial cells. Proc Natl Acad Sci U S A 107: 11543-11548.
